# Supplementary material for: Cardiovascular exercise mitigates inflammation in the early subacute phase of stroke recovery: a secondary analysis of a randomized controlled trial
Source: Brain Behav Immun Health. 2026 Oct 1;58:101375. doi: 10.1016/j.bbih.2026.101375 (PMC13676122; doi:10.1016/j.bbih.2026.101375)

a

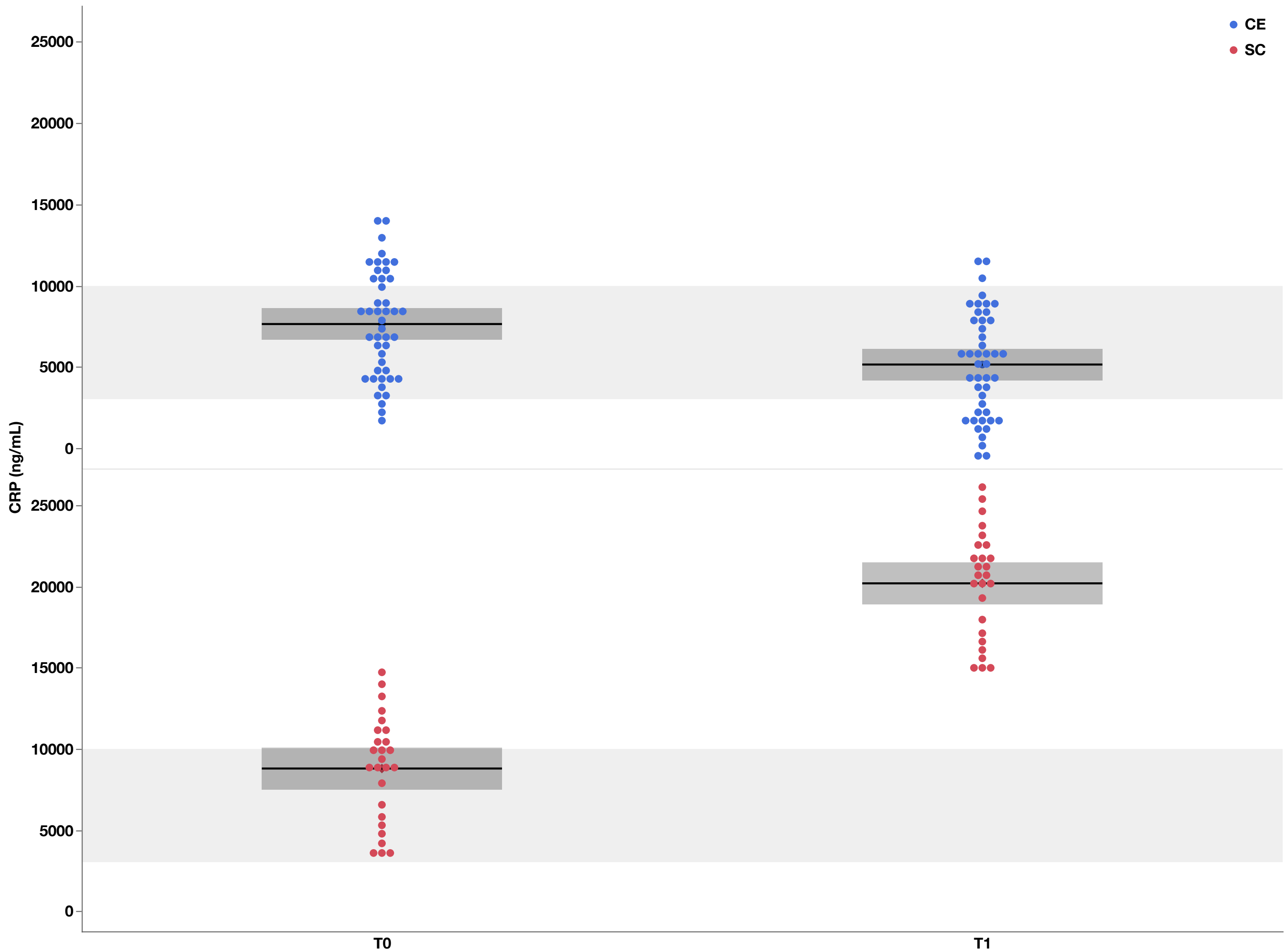

CE
SC
25000
20000
15000
10000
5000
0
25000
20000
15000
10000
5000
0
CRP (ng/mL)
T0
T1

b

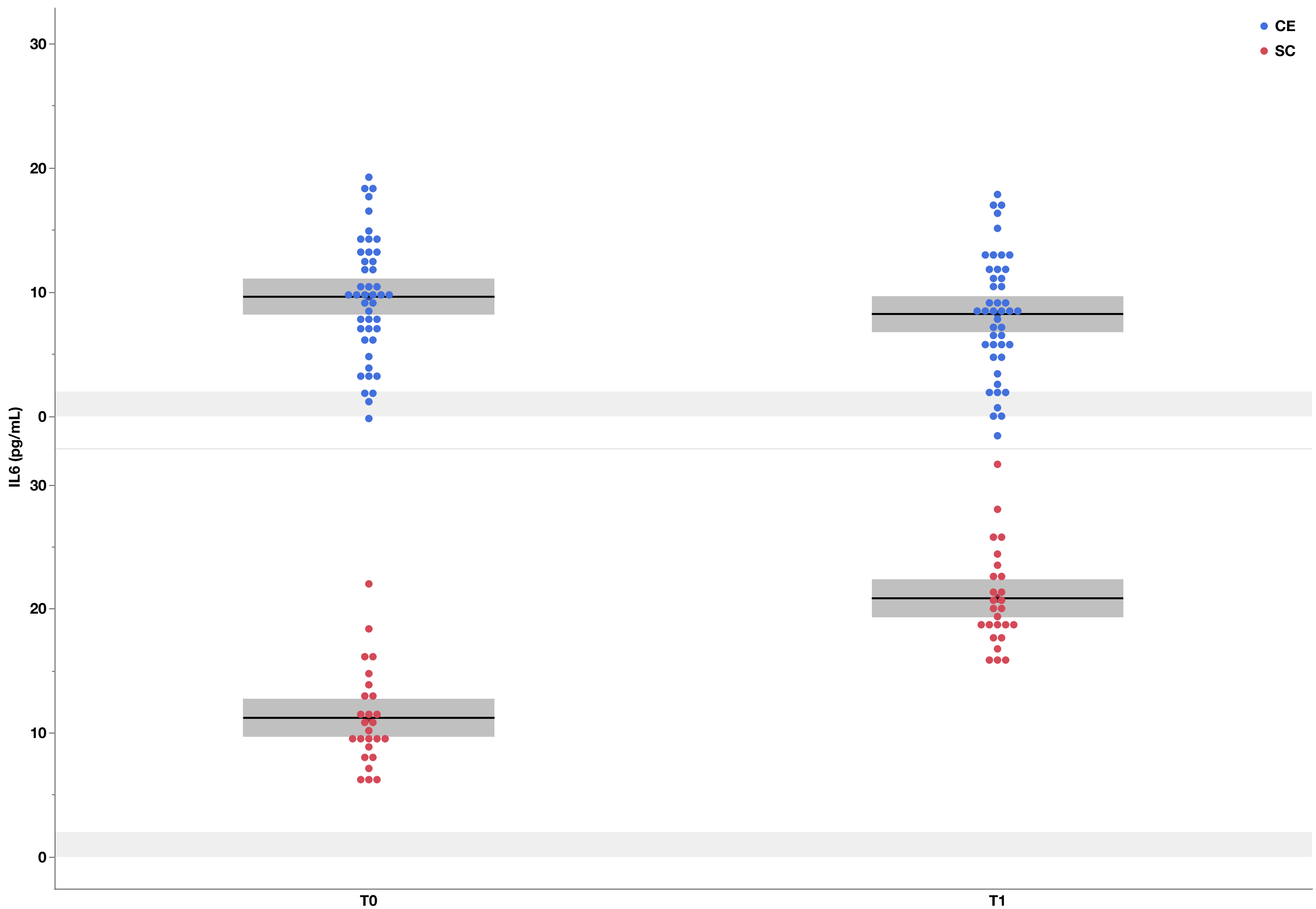

c

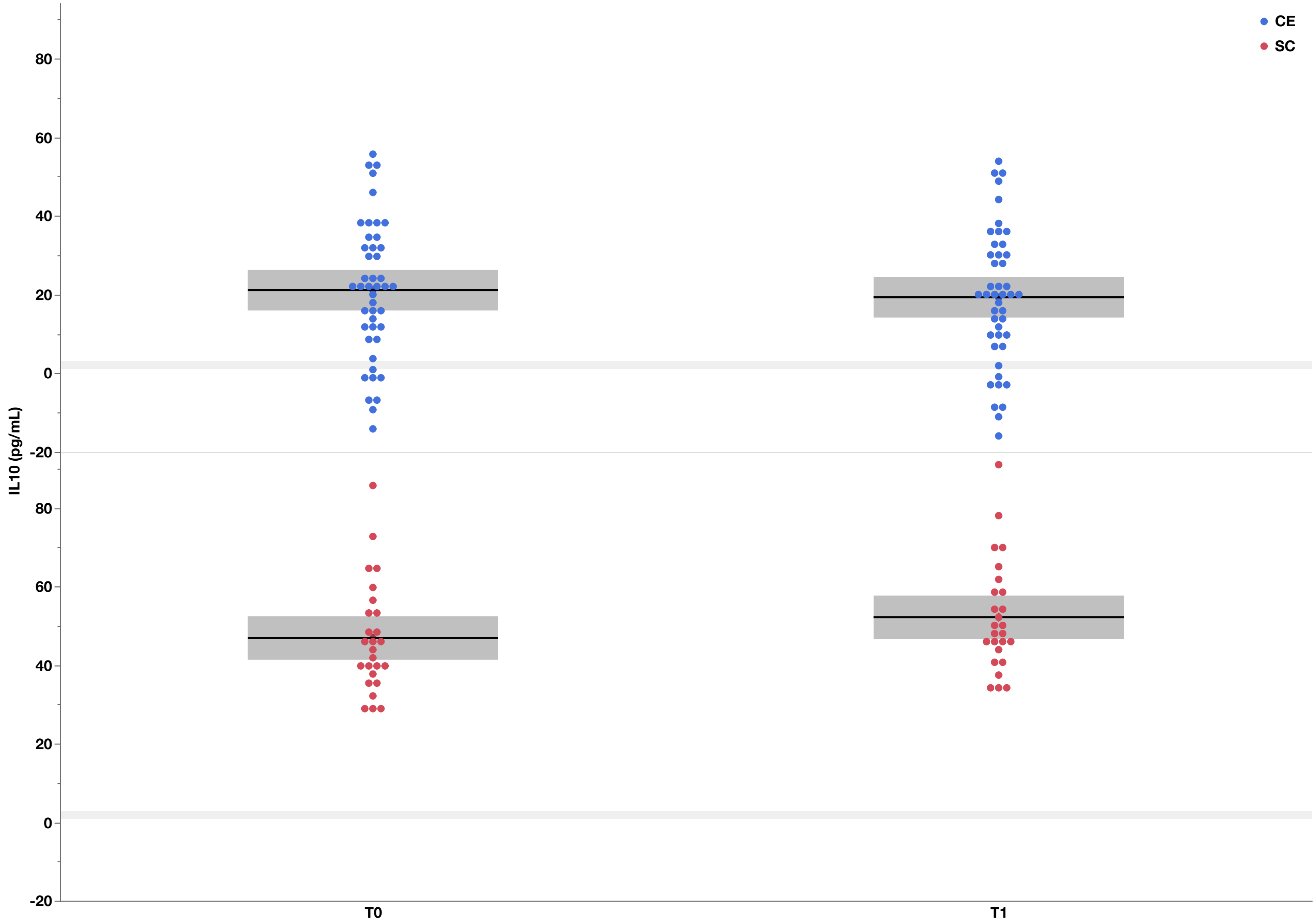

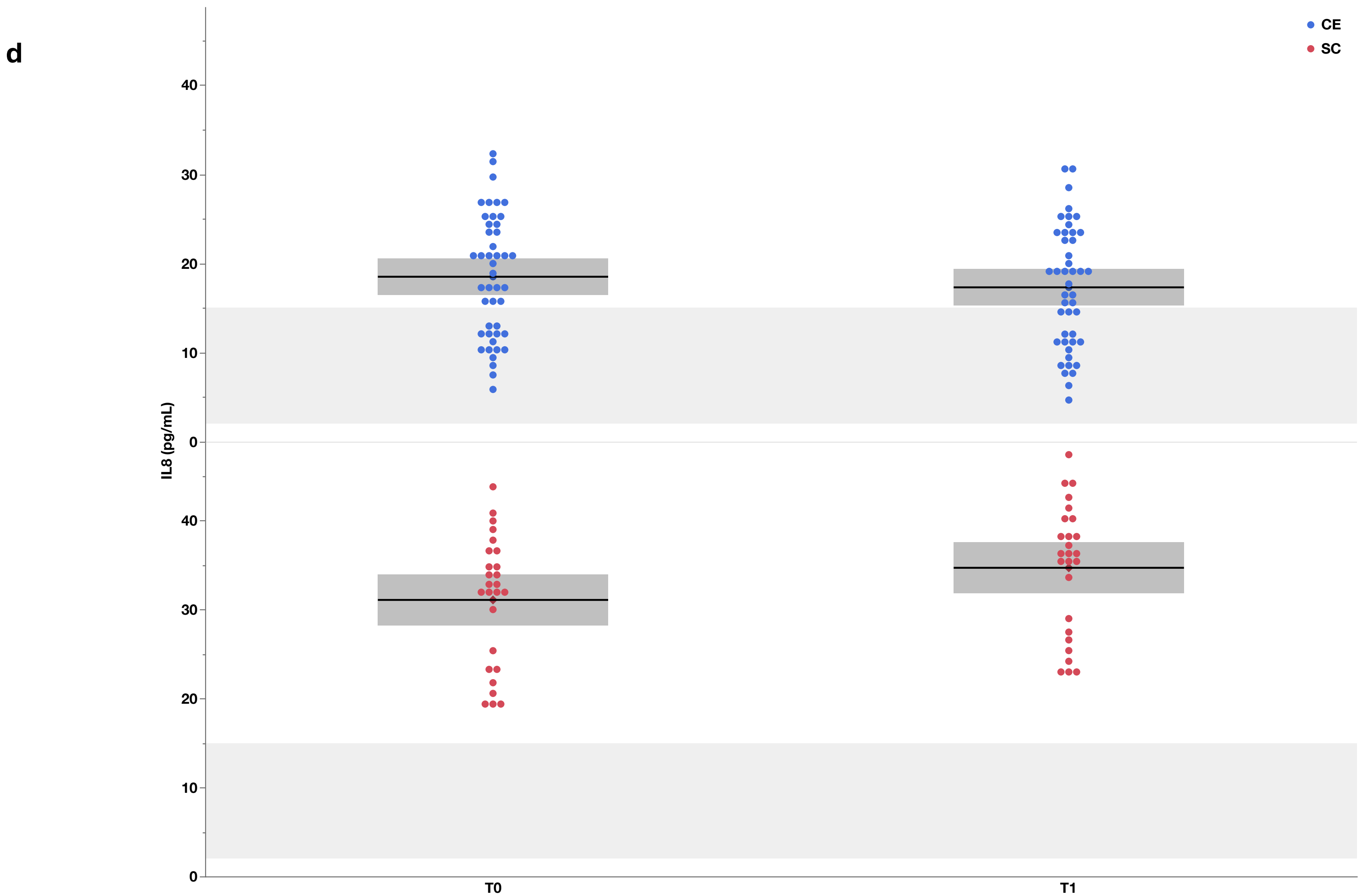
d
CE
SC
IL8 (pg/mL)
40
30
20
10
0
40
30
20
10
0
T0
T1

e

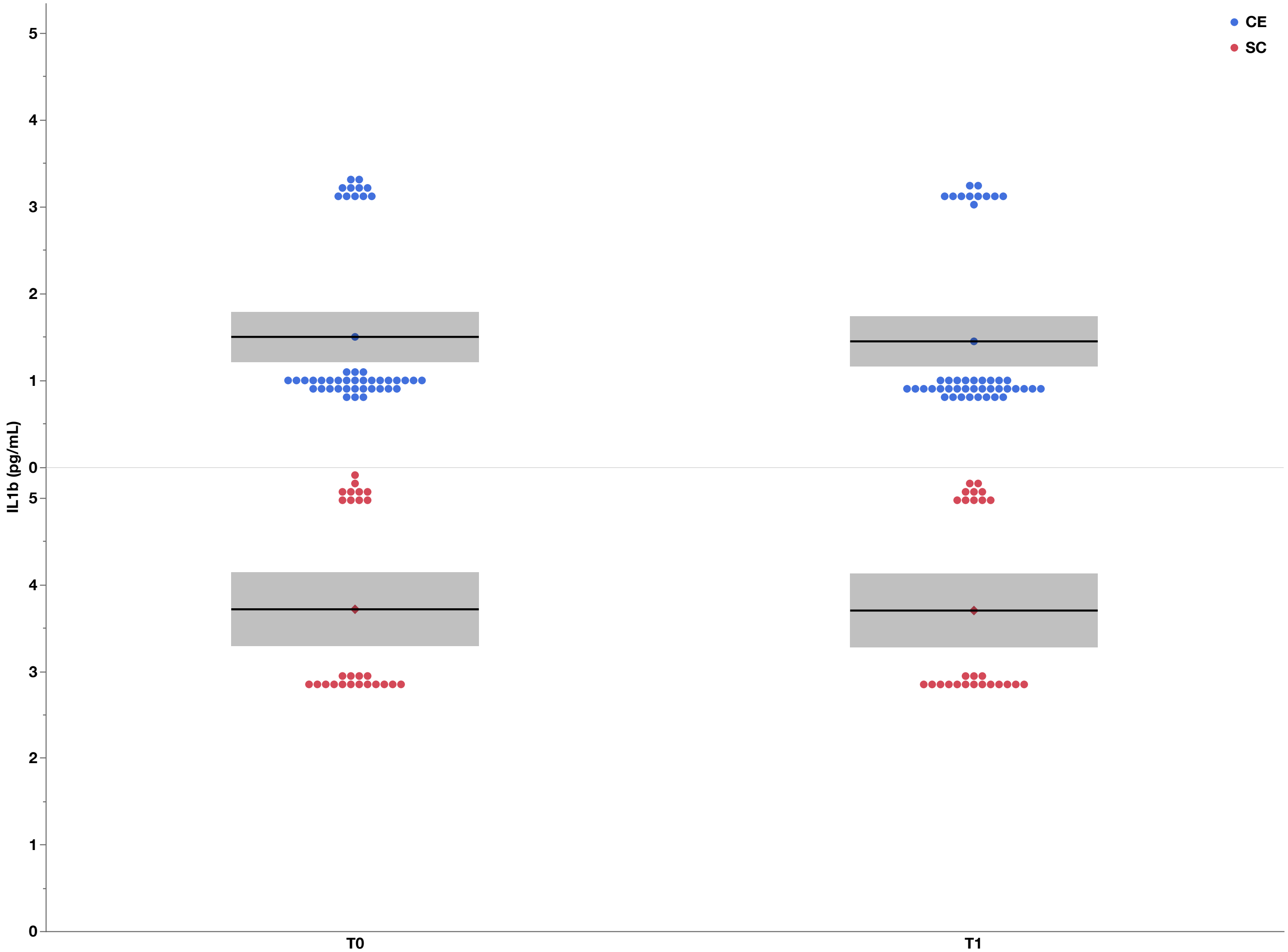

f

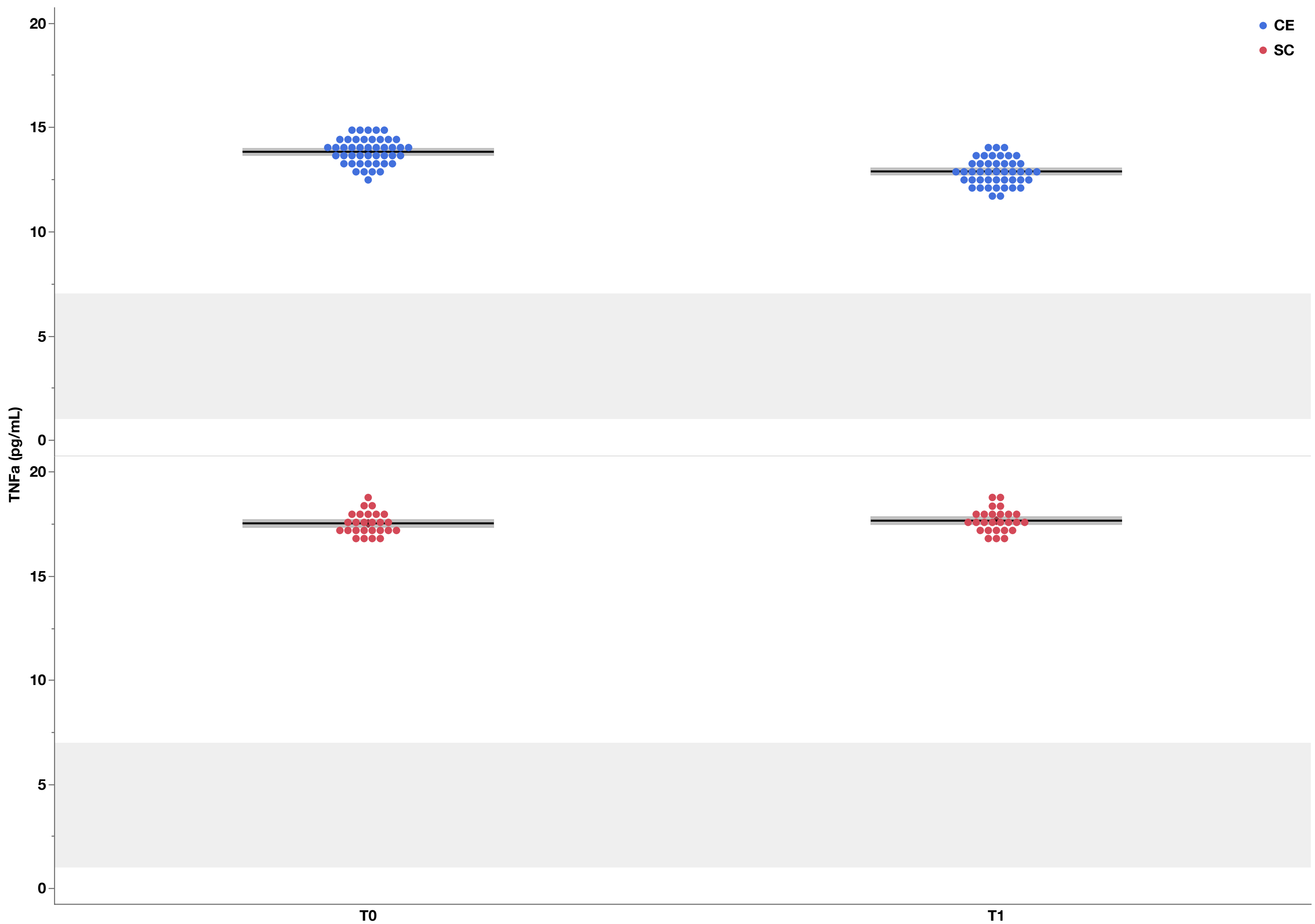

Supplement: Supplementary file 1 [file mmc2.pdf]
