# Supplementary material for: Cardiovascular exercise mitigates inflammation in the early subacute phase of stroke recovery: a secondary analysis of a randomized controlled trial
Source: Brain Behav Immun Health. 2026 Oct 1;58:101375. doi: 10.1016/j.bbih.2026.101375 (PMC13676122; doi:10.1016/j.bbih.2026.101375)
Supplement: Supplementary file 2 [file mmc1.docx]

# Supplementary Tables

**Table S1.** Baseline values of inflammatory mediators for cardiovascular exercise (CE) and standard care (SC) groups.

| **Mediator** | **CE** | **SC** |
| --- | --- | --- |
| CRP, ng/mL | 3591.67 [10137.76] | 4973.38 [8008.72] |
| IL-6, pg/mL | 3.60 [8.46] | 2.68 [4.03] |
| IL-8, pg/mL | 12.68[12.68] | 14.91 [14.28] |
| IL-10, pg/mL | 10.46 [16.13] | 5.24 [8.21] |
| TNF -α pg/mL | 13.12 [8.41] | 16.90 [5.08] |
| IL-1β, pg/mL | 0.8 [1.33] | 0.8 [1.43] |

Values are medians [IQR]

# Table S2. Fixed effects parameters (intention to treat analysis)

| **Parameter** | **β** | **Std Error** | **DF Den** | **95% CI** | **p value** |
| --- | --- | --- | --- | --- | --- |
| **CRP** | | | | | |
| Intercept | 10.0928 | 1.041 | 67.4 | 8.0151, 12.1704 | <0.0001 |
| Group | -0.7454 | 0.3681 | 104.4 | -1.4753, -0.0154 | 0.0455 |
| Time | -0.0519 | 0.263 | 60.2 | -0.5779, 0.4742 | 0.8443 |
| Group×Time | 0.2496 | 0.3282 | 60.0 | -0.4069, 0.9061 | 0.4499 |
| Age | -0.028 | 0.0148 | 65.7 | -0.0575, 0.0015 | 0.0627 |
| Sex | 0.3695 | 0.3293 | 69.1 | -0.2874,1.0263 | 0.2657 |
| **IL-6** | | | | | |
| Intercept | 3.2746 | 1.046 | 65.9 | 1.1863, 5.363 | 0.0026 |
| Group | -0.3553 | 0.3389 | 85.3 | -1.0291, 0.3185 | 0.2974 |
| Time | -0.4067 | 0.1683 | 55.5 | -0.7439, -0.0695 | 0.0190 |
| Group×Time | 0.5848 | 0.2099 | 55.4 | 0.1642, 1.0055 | 0.0073 |
| Age | -0.0283 | 0.0149 | 65.2 | -0.0581, 0.0015 | 0.0620 |
| Sex | 0.2268 | 0.3293 | 66.8 | -0.4305, 0.8841 | 0.4934 |
| **IL-8** | | | | | |
| Intercept | 3.423 | 0.6259 | 67.1 | 2.1737, 4.6723 | <0.0001 |
| Group | -0.4528 | 0.1942 | 75.1 | -0.8395, -0.066 | 0.0224 |
| Time | -0.145 | 0.0626 | 55.1 | -0.2705, -0.0195 | 0.0244 |
| Group×Time | 0.1605 | 0.0781 | 55.1 | 0.0039, 0.317 | 0.0447 |
| Age | -0.0066 | 0.0089 | 66.8 | -0.0245, 0.0112 | 0.4613 |
| Sex | 0.0933 | 0.1966 | 67.5 | -0.2991, 0.4857 | 0.6365 |
| **IL-10** | | | | | |
| Intercept | 4.527 | 1.0191 | 66.5 | 2.4926, 6.5614 | <0.0001 |
| Group | -0.1059 | 0.3177 | 75.8 | -0.7386, 0.5267 | 0.7397 |
| Time | -0.244 | 0.1102 | 54.6 | -0.4648, -0.0232 | 0.0309 |
| Group×Time | 0.2513 | 0.1374 | 54.6 | -0.0241, 0.5267 | 0.0729 |
| Age | -0.0326 | 0.0145 | 66.2 | -0.0617, -0.0036 | 0.0284 |
| Sex | 0.0112 | 0.3202 | 66.9 | -0.628, 0.6503 | 0.9723 |
| **TNF-α** | | | | | |
| Intercept | 2.6581 | 0.5628 | 46.6 | 1.5257, 3.7905 | <0.0001 |
| Group | -0.3339 | 0.1398 | 49.4 | -0.6149, -0.0529 | 0.0208 |
| Time | -0.0188 | 0.0279 | 36.3 | -0.0753, 0.0377 | 0.5037 |
| Group×Time | 0.0845 | 0.0347 | 35.8 | 0.0141, 0.1549 | 0.0200 |
| Age | 0.0004 | 0.0082 | 46.5 | -0.016, 0.0169 | 0.9572 |
| Sex | 0.1042 | 0.1432 | 46.7 | -0.1839, 0.3924 | 0.4703 |
| **IL-1β** | | | | | |
| Intercept | 1.2382 | 0.6908 | 66.9 | -0.1408, 2.6171 | 0.0776 |
| Group | -0.2439 | 0.2085 | 67.6 | -0.6599, 0.1721 | 0.2461 |
| Time | 0.0078 | 0.02 | 54.0 | -0.0323, 0.048 | 0.6976 |
| Group×Time | -0.0076 | 0.025 | 54.0 | -0.0577, 0.0425 | 0.7618 |
| Age | -0.0102 | 0.0099 | 66.9 | -0.0299, 0.0095 | 0.3053 |
| Sex | -0.2582 | 0.2167 | 66.9 | -0.6908, 0.1743 | 0.2376 |

# Table S3. Fixed effects parameters (per-protocol analysis)

| **Parameter** | **β** | **Std Error** | **DF Den** | **95% CI** | **p value** |
| --- | --- | --- | --- | --- | --- |
| **CRP** | | | | | |
| Intercept | 9.5535363 | 1.1266783 | 53.5 | 7.2942175, 11.812855 | <0.0001 |
| Group | -0.675682 | 0.3975614 | 73.9 | -1.46786, 0.1164966 | 0.0934 |
| Time | -0.034922 | 0.2703739 | 54.0 | -0.576989, 0.507145 | 0.8977 |
| Group×Time | 0.1960446 | 0.3372155 | 54.0 | -0.480032, 0.872121 | 0.5634 |
| Age | -0.018662 | 0.016083 | 52.0 | -0.050935, 0.0136109 | 0.2512 |
| Sex | 0.3080263 | 0.3880717 | 52.0 | -0.470697, 1.0867491 | 0.4310 |
| **IL-6** | | | | | |
| Intercept | 3.8680388 | 1.0651664 | 52.7 | 1.7312637, 6.0048138 | 0.0006 |
| Group | -0.365178 | 0.3576919 | 61.8 | -1.08023, 0.3498736 | 0.3113 |
| Time | -0.410823 | 0.1692044 | 54.0 | -0.750058, -0.071589 | 0.0185 |
| Group×Time | 0.5905329 | 0.211035 | 54.0 | 0.1674332, 1.0136326 | 0.0071 |
| Age | -0.038604 | 0.0152672 | 52.0 | -0.06924, -0.007968 | 0.0145 |
| Sex | 0.3133106 | 0.368387 | 52.0 | -0.425912, 1.0525332 | 0.3990 |
| **IL-8** | | | | | |
| Intercept | 3.6133338 | 0.7387364 | 47.1 | 2.1272609, 5.0994068 | 0.0001 |
| Group | -0.418761 | 0.2235643 | 51.1 | -0.867567, 0.0300443 | 0.0668 |
| Time | -0.148301 | 0.06368 | 42.0 | -0.276815, -0.019787 | 0.0248 |
| Group×Time | 0.165089 | 0.0795211 | 49.9 | 0.00536, 0.324818 | 0.0431 |
| Age | -0.010707 | 0.0108542 | 46.8 | -0.032545, 0.0111311 | 0.3290 |
| Sex | 0.1421389 | 0.2336274 | 46.8 | -0.327912, 0.6121895 | 0.5459 |
| **IL-10** | | | | | |
| Intercept | 5.2359721 | 1.0323072 | 52.3 | 3.1647775, 7.3071666 | <0.0001 |
| Group | -0.064249 | 0.3388757 | 56.5 | -0.742972, 0.6144749 | 0.8503 |
| Time | -0.249605 | 0.1104286 | 54.0 | -0.471001, -0.028209 | 0.0279 |
| Group×Time | 0.2631158 | 0.1377287 | 54.0 | -0.013014, 0.5392452 | 0.0614 |
| Age | -0.045759 | 0.0148219 | 52.0 | -0.075501, -0.016017 | 0.0032 |
| Sex | 0.1393133 | 0.3576415 | 52.0 | -0.578347, 0.8569734 | 0.6985 |
| **TNF-α** | | | | | |
| Intercept | 2.4402889 | 0.4971418 | 52.1 | 1.4427381, 3.4378398 | <0.0001 |
| Group | -0.375487 | 0.1608933 | 53.2 | -0.698167, -0.052807 | 0.0234 |
| Time | -0.017833 | 0.0277749 | 54.0 | -0.073518, 0.0378521 | 0.5236 |
| Group×Time | 0.0830595 | 0.0346414 | 54.0 | 0.0136077, 0.1525112 | 0.0200 |
| Age | 0.0043429 | 0.0071454 | 52.0 | -0.009995, 0.0186812 | 0.5460 |
| Sex | 0.1085662 | 0.1724137 | 52.0 | -0.237407, 0.4545396 | 0.5317 |
| **IL-1β** | | | | | |
| Intercept | 1.2618031 | 0.6154942 | 52.0 | 0.0267393, 2.4968669 | 0.0454* |
| Group | -0.15048 | 0.1984832 | 52.4 | -0.548691, 0.2477308 | 0.4518 |
| Time | 0.0078992 | 0.0200263 | 54.0 | -0.032251, 0.0480494 | 0.6948 |
| Group×Time | -0.007298 | 0.0249771 | 54.0 | -0.057374, 0.042778 | 0.7713 |
| Age | -0.009743 | 0.0088488 | 52.0 | -0.027499, 0.0080133 | 0.2759 |
| Sex | -0.325958 | 0.2135146 | 52.0 | -0.754406, 0.1024904 | 0.1329 |

**Table S4**. Associations between inflammatory mediators at baseline for both groups.

|  | **CRP** | **IL-6** | **IL-8** | **IL-10** | **TNF-α** | **IL-1β** |
| --- | --- | --- | --- | --- | --- | --- |
| CRP | – |  |  |  |  |  |
| IL-6 | 0.13 | – |  |  |  |  |
| IL-8 | 0.01 | 0.42 | – |  |  |  |
| IL-10 | -0.05 | 0.68 | 0.32 | – |  |  |
| TNF-α | 0.05 | -0.19 | 0.39 | -0.33 | – |  |
| IL-1β | -0.01 | 0.24 | 0.36 | 0.13 | 0.00 | – |

Values are Spearman’s correlation coefficients (*r*^2^). Values in red denote statistically significant correlations (p<0.05)

**Table S5.** Associations between inflammatory mediators pre-post intervention (Δ=concentration T1- concentration T0) for cardiovascular exercise (CE) and standard care (SC) groups.

| **CE** | | | | | | |
| --- | --- | --- | --- | --- | --- | --- |
|  | **CRP** | **IL-6** | **IL-8** | **IL-10** | **TNF-α** | **IL-1β** |
| CRP | – |  |  |  |  |  |
| IL-6 | 0.23 | – |  |  |  |  |
| IL-8 | 0.10 | 0.55 | – |  |  |  |
| IL-10 | -0.03 | 0.59 | 0.24 | – |  |  |
| TNF-α | 0.01 | 0.37 | 0.13 | 0.29 | – |  |
| IL-1β | -0.16 | 0.43 | 0.35 | 0.30 | -0.05 | – |
| **SC** | | | | | | |
|  | **CRP** | **IL-6** | **IL-8** | **IL-10** | **TNF-α** | **IL-1β** |
| CRP | – |  |  |  |  |  |
| IL-6 | 0.22 | – |  |  |  |  |
| IL-8 | -0.25 | 0.21 | – |  |  |  |
| IL-10 | 0.05 | 0.49 | 0.53 | – |  |  |
| TNF-α | 0.02 | -0.06 | 0.70 | 0.54 | – |  |
| IL-1β | 0.00 | 0.42 | 0.20 | 0.68 | 0.10 | – |

Values are Spearman’s correlation coefficients. Values in red denote statistically significant correlations (p<0.05)

**Table S6.** Associations between inflammatory mediators pre-post intervention (Δ=concentration T1- concentration T0) and changes in cardio-respiratory fitness (VO_2_ peak), cognition (MoCA), degree of neurological (NIHSS) and motor (Fugl-Meyer) deficit for cardiovascular exercise (CE) and standard care (SC) groups.

| **CE** | | | | | | |
| --- | --- | --- | --- | --- | --- | --- |
|  | **CRP** | **IL-6** | **IL-8** | **IL-10** | **TNF-α** | **IL-1β** |
| VO_2peak_ | -0.03 | -0.35 | -0.16 | -0.09 | -0.01 | 0.90 |
| MoCA | 0.02 | 0.15 | -0.19 | 0.17 | -0.09 | -0.49 |
| NIHSS | -0.19 | 0.11 | -0.30 | 0.17 | -0.01 | 0.34 |
| Fugl-Meyer | 0.15 | -0.52 | -0.16 | -0.57 | 0.36 | 0.15 |
| **SC** | | | | | | |
|  | **CRP** | **IL-6** | **IL-8** | **IL-10** | **TNF-α** | **IL-1β** |
| VO_2peak_ | -0.40 | 0.40 | 0.02 | -0.14 | -0.12 | -0.70 |
| MoCA | 0.10 | -0.24 | -0.42 | -0.35 | -0.17 | -0.82 |
| NIHSS | -0.89 | -0.42 | 0.25 | 0.19 | -0.31 | 0.11 |
| Fugl-Meyer | 0.16 | 0.33 | -0.12 | -0.06 | 0.37 | 0.10 |

Values are Spearman’s correlation coefficients. Values in red denote statistically significant correlations (p<0.05)

**Supplementary Figures Captions**

**Figure S1 a-f**. Least square mean estimates plots showing changes in the concentration of the inflammatory mediators CRP, IL-6, IL-8, IL-10, TNF-α and IL-1β for CE and SC from T0 to T1. Values are non-log-transformed predicted mean estimates with 95% CIs. Shaded grey areas represent reference concentration ranges obtained from studies in which the concentration of these mediators was assessed in serum. Ranges for IL-1β are not provided due to the lack of consistent data. These ranges are only approximations and should be interpreted cautiously.
